# Social isolation, cognitive reserve, and cognition in healthy older people

**DOI:** 10.1371/journal.pone.0201008

**Published:** 2018-08-17

**Authors:** Isobel E. M. Evans, David J. Llewellyn, Fiona E. Matthews, Robert T. Woods, Carol Brayne, Linda Clare, on behalf of the CFAS-Wales research team

**Affiliations:** 1 Centre for Research in Ageing and Cognitive Health (REACH), School of Psychology, University of Exeter, Exeter, United Kingdom; 2 University of Exeter Medical School, Exeter, United Kingdom; 3 Institute of Health and Society, Faculty of Medicine, Newcastle University, Newcastle, United Kingdom; 4 MRC Biostatistics Unit, Institute of Public Health, University of Cambridge, Cambridge, United Kingdom; 5 Dementia Services Development Centre Wales, School of Healthcare Sciences, Bangor University, Bangor, United Kingdom; 6 Institute of Public Health, University of Cambridge, Cambridge, United Kingdom; 7 PenCLAHRC, Institute of Health Research, University of Exeter Medical School, Exeter, United Kingdom; 8 Centre for Research Excellence in Promoting Cognitive Health, Australian National University, Canberra, Australia; Nathan S Kline Institute, UNITED STATES

## Abstract

There is evidence to suggest that social isolation is associated with poor cognitive health, although findings are contradictory. One reason for inconsistency in reported findings may be a lack of consideration of underlying mechanisms that could influence this relationship. Cognitive reserve is a theoretical concept that may account for the role of social isolation and its association with cognitive outcomes in later life. Therefore, we aimed to examine the relationship between social isolation and cognition in later life, and to consider the role of cognitive reserve in this relationship. Baseline and two year follow-up data from the Cognitive Function and Ageing Study–Wales (CFAS-Wales) were analysed. Social isolation was assessed using the Lubben Social Network Scale-6 (LSNS-6), cognitive function was assessed using the Cambridge Cognitive Examination (CAMCOG), and cognitive reserve was assessed using a proxy measure of education, occupational complexity, and cognitive activity. Linear regression modelling was used to assess the relationship between social isolation and cognition. To assess the role of cognitive reserve in this relationship, moderation analysis was used to test for interaction effects. After controlling for age, gender, education, and physically limiting health conditions, social isolation was associated with cognitive function at baseline and two year follow-up. Cognitive reserve moderated this association longitudinally. Findings suggest that maintaining a socially active lifestyle in later life may enhance cognitive reserve and benefit cognitive function. This has important implications for interventions that may target social isolation to improve cognitive function.

## Introduction

Cognitive health is an important aspect of healthy ageing. As people get older, they may experience subtle changes in their cognitive ability, a process referred to as ‘cognitive ageing’ [1]. It is widely thought that while cognitive ageing is a normal part of healthy ageing, more significant changes in cognition are not [2, 3]. Variation is observed in the trajectories of cognitive ageing across older people [4]. While some individuals retain a high level of cognitive ability from mid- to late- life, others may experience decline [5]. A decline in cognitive function can be detrimental to the independence, wellbeing, and quality of life of older people [6, 7]. Understanding the mechanisms underlying differences in cognitive ageing is important to reduce the impact of poor cognitive function in later life.

Cognitive reserve is a theoretical concept that can account for the differences observed in late-life cognitive trajectories. The theory suggests that individuals differ in their degree of resilience against age-related brain pathology and hence may show differences in cognitive function in relation to an equivalent level of pathology [8, 9]. These differences are linked to the ability of an individual to recruit protective mechanisms associated with cognitive abilities built up over the lifespan, and actively compensate for damage caused by pathology [10]. Observational and some experimental evidence suggests that reserve can be built up through a combination of experiences across the lifespan, such as physical exercise, education, occupation, and participation in social and cognitively stimulating activities. These experiences may create a buffer against cognitive decline by enhancing brain processes such as neural connectivity and hence cognitive ability. This might then protect the individual against the effects of disease pathology in the first instance, and also compensate for damage and recruit alternative neural pathways when required. This may reduce or delay the extent of impairment experienced and protect against the expression of pathological processes [11].

Compared to other lifestyle factors that may build cognitive reserve, the association between social connections and cognition is less well understood. Having a range of good social connections has been identified as an important aspect of successful ageing [12] and is associated with lower mortality rates [13, 14], better health outcomes [15, 16], and higher reported wellbeing [17, 18] and quality of life [19, 20]. There is also evidence to suggest that social isolation may be associated with poor cognitive health [21]. Social isolation can be defined as a state in which an individual has a minimal number of social contacts and lacks engagement with others and the wider community [22]. Based on the cognitive reserve theory, social integration would provide mental stimulation through complex communication and interaction with others [23, 24]. In contrast, being isolated would not provide this stimulation and hence may not build reserve.

There is some evidence that social isolation is associated with poorer cognitive outcomes [25–28]. However, findings are inconsistent and some studies report conflicting relationships [26, 28, 29]. Inconsistency in results assessing the relationship between social isolation and cognitive function may be attributed to conceptual and methodological challenges associated with defining and measuring social isolation. There is no consistent definition used within the literature and this inconsistency is reflected in the measures selected to assess social isolation. Some authors include other indicators in definitions and measures of isolation, such as being unmarried and living alone [26, 27] or social support [26]. Including these indicators in measures of isolation reduces the validity of findings, as they do not necessarily reflect isolation. Some studies use standardised measures of social isolation, such as the Lubben Social Network Scale–6 (LSNS-6) [25, 29], whereas others compose measures that capture features of social isolation, such as social networks or social engagement [27, 28]. Likewise, the assessment of cognitive outcomes varies across studies, with some using domain-specific measures of cognitive ability such as attention, or executive function [25, 27], and others using more comprehensive global measures of cognition [29], or dementia diagnosis [26, 28]. Variation in approaches to assessing and defining social isolation and cognitive outcomes may contribute to inconsistency and complicate the interpretation of empirical findings.

Given that current findings are conflicting, this study aims to assess the relationship between social isolation and cognitive function. This relationship will be examined using baseline and two-year follow up data from the Cognitive Function and Ageing Study–Wales (CFAS-Wales). Although previous work suggests that cognitive reserve may be important in this association, to our knowledge, this has not been explored. One previous study has assessed the moderating effect of education on the association between social isolation and cognitive function [27]. The authors reported a moderating effect for delayed recall, but not for immediate recall or verbal fluency [27]. Education alone is not a comprehensive indication of cognitive reserve and it is suggested that multiple proxy indicators representing reserve at different stages of life are preferable [30, 31]. Therefore, we aim to consider whether cognitive reserve moderates the association between social isolation and cognitive function, using a comprehensive measure of cognitive reserve.

## Method

### Design

The relationship between social isolation, cognitive reserve, and cognitive function was examined using data from the Cognitive Function and Ageing Study–Wales (CFAS-Wales). CFAS-Wales is a longitudinal study of people aged 65 and over, conducted across two locations in Wales, one rural (Gwynedd and Ynys Môn) and one urban (Neath Port Talbot). Participants were assessed at baseline and again two years later. CFAS-Wales aims to investigate physical and cognitive health in later life and to examine environmental factors that may contribute to activity and participation in community and civic life. The North Wales research ethics committee granted ethical approval for data collection (Ref No: 10/WNo01/37; IRAS Project No:40092).

### Study population

People aged 65 and over were randomly sampled from general practice lists between 2011 and 2013. Participants were stratified by age to ensure a representative sample across two age groups (65–74 and ≥75). People who consented to take part completed an interview at home. Interviews were conducted by research assistants who had completed training provided by staff at the co-ordinating centre in Cambridge and participants could choose whether they wanted their interview conducted in English or Welsh. Baseline interviews took place between 2011 and 2013 and follow-up interviews were completed two years later, between 2013 and 2015.

The present study uses baseline data which were collected for 3,593 people and follow-up data which were collected for 2,236. We excluded people at baseline with cognitive impairment (MMSE score ≤25; N = 908) or with an AGECAT classification of dementia (N = 185) to reduce the risk of reverse causation. The AGECAT is a diagnostic algorithm embedded in the CFAS-Wales interview that assesses symptoms to determine whether a person has a healthy diagnosis, or a diagnosis of dementia, depression, or anxiety [32]. Given that depression is also associated with cognitive impairment, people with an AGECAT classification of depression (N = 333) were also excluded, as were people living in an institution (N = 95) as it is considered that the experience of social isolation will differ between community and residential settings. Participants with missing data on the measures used at baseline (N = 146) and follow-up (N = 700) were also excluded. This gave a final sample of 2,224 for cross-sectional analyses and 1,524 for longitudinal analyses. A comparison of participants that were included for cross-sectional analyses, but excluded for longitudinal analyses because of missing data at follow-up, can be found in “S1 Table”. Those who were excluded were older, had a poorer CAMCOG score at baseline, had fewer years of education, engaged in less cognitive activity, had a lower occupational complexity, had a lower cognitive reserve score, were more socially isolated, had poorer eyesight, and required significantly more help with daily tasks, but were no more likely to be women or have problems with hearing.

### Measures

#### Cognitive function

Cognitive function was assessed using the Cambridge Cognitive Examination (CAMCOG) [33]. The CAMCOG is a standardised measure that consists of 67 items, assessing cognitive function along eight subscales, including orientation, comprehension, expression, memory (remote, recent, and learning), attention and calculation, praxis, abstract thinking, and perception. Total scores range from 0–107 and a lower score indicates poor cognitive function. Both baseline and follow-up CAMCOG scores are used in the analyses.

#### Social isolation

Social isolation was measured in CFAS-Wales using the Lubben Social Network Scale–6 (LSNS-6) [34]. The LSNS-6 is a standardised measure of social isolation, constructed of three sets of questions that assess family ties, and a set of three comparable questions assessing non-kinship ties. The three items assess the number of relatives/ friends the participant sees or hears from at least once a month, could call on for help, and can speak with about private matters. Responses are collected using a six category response, in which the participant indicates the number of relatives/ friends available. Response scores range from 0 (no relatives/ friends) to 5 (nine or more relatives/ friends). The overall scores for each six questions are summed and range from 0–30, with higher scores indicating lower social isolation. A score of ≤12 may be taken to indicate the presence of social isolation [34]. Baseline LSNS-6 scores were used for all analyses.

#### Cognitive reserve

Cognitive reserve was measured by combining three proxy indicators: education, occupational complexity, and cognitive activity at baseline. Education was recorded as the number of years in full time education. Occupational complexity was assessed using social class and the social economic group and complexity of the participant’s main employment. These were combined to create an occupational complexity score ranging from 1 (lower class and less complex occupations, e.g. cleaner) to 14 (high class and complex occupations, e.g. doctor or lawyer). Cognitive activity was assessed by seven questions asking about engagement in a range of cognitive activities (including listening to the radio, reading a newspaper, magazine, book, playing games such as card or chess, crosswords, and puzzles). Responses were recorded on a 5-point Likert scale (once a year or less, several times a year, several times a month, several times a week, or everyday/ almost every day) and higher scores indicate greater cognitive activity.

Scores for each proxy indicator were weighted based on the interquartile range to ensure that each component contributed equally to determining the cognitive reserve score. This gave the following formula: cognitive reserve score = (2.33 x education) + (1.40 x occupational complexity) + (1 x cognitive activity). A higher score indicates higher levels of cognitive reserve.

#### Covariates

Several covariates were controlled for in the analyses, including age (years), gender, and education (years) at baseline, which are established covariates of late-life cognition [35–37]. Education was not controlled for in analyses that assessed cognitive reserve. Sensory problems (hearing and eyesight) at baseline were also controlled for as these problems may reduce an individual’s ability to be socially engaged and hence contribute to an increased level of social isolation [38, 39]. Participants were asked whether hearing or sight problems limit day-to-day activities (yes/ no). If hearing or eyesight problems are not problematic because the participant wears a hearing aid or glasses then this is rated as no. Finally, the participants’ ability to complete daily tasks alone (such as housework, getting dressed, getting up and down stairs, carrying things, etc.) at baseline was controlled for. Participants were asked if they receive any help with day-to-day activities and respond yes/ no. Being unable to complete such tasks may indicate limitations in physical ability and mobility, which may influence ability to be socially engaged, and hence increase level of social isolation [40].

### Statistical analysis

#### Descriptive data

All analyses were conducted in Stata version 15.0. Descriptive information is reported for the overall sample at baseline, and separately for those who are socially isolated (score of ≤12 on the LSNS-6) and those who are not isolated.

CAMCOG scores were normally distributed at baseline (skewness of -.84 and kurtosis of 4.48) and follow-up (skewness of -1.16 and kurtosis of 5.44). The LSNS-6 was also normally distributed at baseline (skewness of -.13 and kurtosis of 2.61).

#### Regression analyses

For the baseline data, linear regression modelling was used to assess the association between social isolation, as determined by scores on the LSNS-6, and cognitive function, adjusting for covariates. This approach was also used to assess the relationship between social isolation at baseline and cognitive change over two year follow-up. For all longitudinal analyses, a cognitive change score was calculated by subtracting the CAMCOG score at baseline from the CAMCOG score at follow-up. Each participant’s cognitive change score was then standardised by the standard deviation value of the baseline CAMCOG score.

In step one of the model, unadjusted effects for the association between social isolation and cognition are reported. Step two adjusts for age, gender, and education. Step three adjusts for age, gender, education, physically limiting health conditions (eyesight and hearing), and help with daily activities. Adjusted R^2^ values were reported for regression models to indicate the proportion of variance explained by variables in the model. Regression coefficients were also reported, along with 95% confidence intervals. All measures were standardised to provide comparable coefficients.

#### Moderation analyses

Moderation analyses were conducted to determine whether cognitive reserve moderates the association between social isolation and cognitive function or cognitive change. These analyses tested for an interaction between social isolation and the cognitive reserve score and were adjusted for all covariates (including baseline age, gender, eyesight and hearing, and help with daily activities) except for education, as this is a component of the cognitive reserve score.

## Results

### Descriptive data

The baseline characteristics for the sample are summarised in Table 1. The mean age of participants was 73.47 years and 50.67% of participants were women. At baseline, 601 (27.02%) of participants were classed as isolated by the LSNS-6. People who were isolated were older, more likely to be men, had a poorer CAMCOG score, fewer years of education, participated in fewer cognitive activities, and had lower cognitive reserve scores. There was little change in the mean CAMCOG scores at baseline (*M* = 93.48, *SD* = 5.35) and two year follow-up (*M* = 93.73, *SD* = 6.08) across the total sample.

**Table 1:** Summary of baseline characteristics of participants in CFAS-Wales.

	Total sample (N = 2,224)		Not socially isolated (N = 1,623)	Socially isolated (N = 601)	
Variable	*M* (SD) or *N* (%)	Range	*M* (SD) or *N* (%)	*M* (SD) or *N* (%)	*t*(df) or X^2^(df), *p*
Age (years), *M* (SD)	73.47 (6.28)	65–100	73.09 (6.00)	74.50 (6.91)	t(1, 2222) = -4.72 *p* < .001
Gender, *N* (%)					
Men	1,097 (49.33)		766 (47.20)	331 (55.07)	X^2^(1) = 10.89
Women	1,127 (50.67)		857 (52.80)	270 (44.93)	*p* < .001
Baseline CAMCOG score, *M* (SD)	93.48 (5.35)	63–105	93.84 (5.21)	92.51 (5.62)	t(1, 2222) = 5.24 *p* < .001
Education (years), *M* (SD)	12.05 (2.79)	1–30	12.17 (2.85)	11.73 (2.59)	t(1, 2222) = 3.31 *p* < .001
Cognitive activity, *M* (SD)	21.33 (5.19)	7–34	21.90 (5.05)	19.78 (5.24)	t(1, 2222) = 8.72 *p* < .001
Occupation complexity, *M* (SD)	8.11 (3.32)	1–14	8.18 (3.33)	7.90 (3.31)	t(1, 2222) = 1.82 *p* = .07
Cognitive reserve score, *M* (SD)	60.77 (11.40)	33.53–109.30	61.72 (11.48)	58.17 (10.77)	t(1, 2222) = 6.58 *p* < .001
LSNS-6, *M* (SD)	16.14 (5.76)	0–30	18.83 (3.97)	8.85 (2.74)	t(1, 2222) = 56.82 *p* < .001
Health conditions, *N* (%)^a^					
Hearing	629 (28.28)		449 (27.66)	180 (29.95)	X^2^(1) = 1.13 *p* = .29
Eyesight	311 (13.98)		213 (13.12)	98 (16.31)	X^2^(1) = 3.69 *p* = .06
Require help with daily tasks, *N* (%)	637 (28.64)		452 (27.85)	185 (30.78)	X^2^(1) = 1.85 *p* = .17

Notes

^a^ Number and percentage of people who have these health conditions and rate these conditions as physically limiting.

### Association between social isolation and cognitive function

#### Baseline

A linear regression was conducted to assess the cross-sectional association between social isolation and cognitive function (Table 2). Social isolation was significantly associated with cognition, adjusted R^2^ = .02, *F*(1, 2222) = 35.99, *p* < .001. After adjusting for all covariates, the association remained significant, adjusted R^2^ = .17, *F*(7, 2216) = 64.67, *p* < .001. This model suggested that people who are less socially isolated had better CAMCOG scores and the model explained about 17% of the variance in CAMCOG scores.

**Table 2:** Cross sectional association between social isolation and cognition.

	Model 1 *B* (95% CI) *p*	Model 2 *B* (95% CI) *p*	Model 3 *B* (95% CI) *p*
**Social isolation**	.07 (.05, .10) < .001	.05 (.02, .07) < .001	.04 (.02, .07) < .001
**Age**	-	-.03 (-.03, -.02) < .001	-.02 (-.03, -.02) < .001
**Gender**	-	-.08 (-.12, -.04) < .001	-.08 (-.12, -.03) < .001
**Education**	-	.04 (.03, .05) < .001	.04 (.03, .05) < .001
**Eyesight**	-	-	-.11 (-.17, -.05) < .001
**Hearing**	-	-	-.06 (-.11, -.01) .02
**Help with daily activity**	-	-	-.07 (-.12, -.02) .01

Notes: Model 1: unadjusted; Model 2: adjusted for age, gender, and years of education; Model 3: adjusted for age, gender, education, physically limiting health conditions (eyesight and hearing), and help with daily activities.

We ran a linear regression for men and women separately to consider gender differences, controlling for all covariates, except for gender. We found that the association between social isolation and cognitive function was significant for women, adjusted R^2^ = .17, F(6, 1120) = 38.41, *p* < .001, but not men, adjusted R^2^ = .09, F(6, 1090) = 35.85, *p* = .10. There was a significant interaction between social isolation and gender, adjusted R^2^ = .17, F(8, 2215) = 57.23, *p* = .04.

We conducted further regression analyses to determine whether social isolation was more associated with any specific cognitive domains assessed by the CAMCOG (see “S2 Table”). Social isolation was significantly associated with orientation, expression, praxis, and perception, but not with comprehension, memory, attention and calculation, or abstract thinking.

#### Longitudinal

A linear regression was conducted to assess the association between social isolation at baseline and cognitive change over two year follow-up (Table 3). Social isolation was significantly associated with cognitive change, adjusted R^2^ = .01, *F*(1, 1522) = 8.92, *p* = .003. After adjusting for covariates, the association between social isolation and cognitive change remained significant, adjusted R^2^ = .05, *F*(7, 1516) = 11.95, *p* < .001. This suggests that people who are less socially isolated show less decline in CAMCOG scores over two year follow-up. As there was little cognitive change observed in the sample, a logistic regression was conducted as a sensitivity analysis and can be found in “S3 Table”.

**Table 3:** Longitudinal association between social isolation and cognitive change score.

	Model 1 *B* (95% CI) *p*	Model 2 *B* (95% CI) *p*	Model 3 *B* (95% CI) *P*
**Social isolation**	.07 (.02, .12) .003	.05 (0, .10) .03	.05 (.01, .10) .03
**Age**	-	-.03 (-.04, -.02) < .001	-.03 (-.04, -.02) < .001
**Gender**	-	-.07 (-.16, .02) .11	-.06 (-.15, .03) .19
**Education**	-	.01 (0, .03) .17	.01 (0, .03) .18
**Eyesight**	-	-	-.02 (-.15, .12) .78
**Hearing**	-	-	.09 (-.01, .19) .09
**Help with daily activity**	-	-	-.03 (-.14, .07) .56

Notes: Model 1: unadjusted; Model 2: adjusted for age, gender, and years of education; Model 3: adjusted for age, gender, education, physically limiting health conditions (eyesight and hearing), and help with daily activities.

To consider differences in gender, we ran a linear regression for men and women separately, controlling for all covariates, except for gender. We found that the association between social isolation and cognitive function was significant for women, adjusted R^2^ = .08, F(6, 759) = 11.57, *p* = .02, but not men, adjusted R^2^ = .03, F(6, 751) = 4.65, *p* = .39. When testing for an interaction between social isolation and gender there was no significant difference, adjusted R^2^ = .05, F(8, 1515) = 10.75, *p* = .13.

We conducted further regression analyses to determine whether social isolation was more associated with any specific cognitive domains assessed by the CAMCOG using longitudinal data (see “S4 Table”). Social isolation was significantly associated with comprehension, but not with orientation, expression, memory, attention and calculation, praxis, abstract thinking, and perception.

### Association between social isolation, cognitive reserve, and cognitive function

#### Baseline

A moderation analysis was conducted to assess whether cognitive reserve score moderated the cross-sectional relationship between social isolation and cognition, controlling for all covariates. The interaction term between social isolation and the cognitive reserve score did not explain a significant increase in cognitive function (.01; 95% CI: -.01, .04). None of the individual components of the cognitive reserve score (education, occupational complexity, and cognitive activity) significantly moderated the relationship between social isolation and cognitive function.

#### Longitudinal

A moderation analysis was conducted to assess whether cognitive reserve moderated the longitudinal association between social isolation and cognitive change over two year follow-up, controlling for all covariates. The interaction term between social isolation and cognitive reserve score significantly moderated the cognitive change score (.05; 95% CI: .10, 0).

Post-hoc analyses were conducted to examine the contribution of each component of the cognitive reserve score to determine whether any of the individual components were contributing to the relationship more. The interaction terms between social isolation and education (-.04; 95% CI: -.08, .01) and social isolation and cognitive activity (-.01; 95% CI: -.06, .03) did not explain a significant increase in cognitive function after adjusting for covariates. However, the interaction terms between social isolation and occupational complexity were significant (-.05; 95% CI: -.10, -.01,).

**Fig 1:**
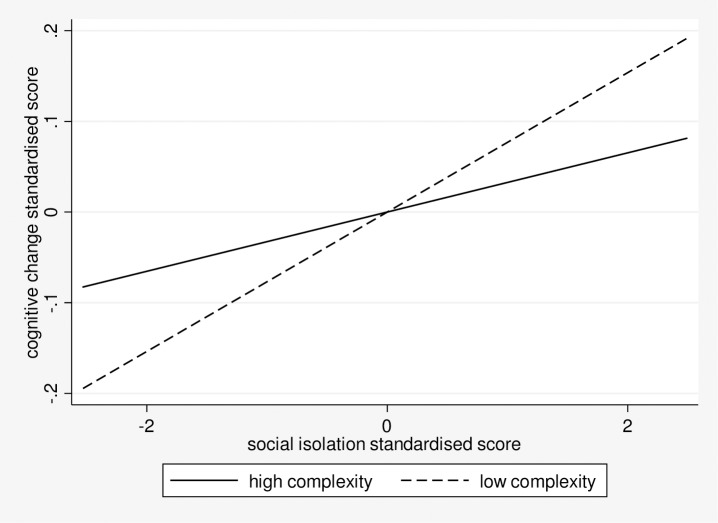
The association between social isolation and cognitive change by high and low occupational complexity groups.

To investigate the relationship between social isolation, occupational complexity, and cognitive change further, participants were separated into two groups: high and low occupational complexity. Regression analyses to assess the association between social isolation and cognitive function were conducted for each group separately, controlling for all covariates. The association between social isolation and cognitive change was non-significant for those with high occupational complexity (.03; 95% CI: -.02, .09), but was significant for those with low occupational complexity (.08; 95% CI: 0, .15; “Fig 1”).

## Discussion

This study aimed to assess the relationship between social isolation and cognitive function, and to consider the role of cognitive reserve in this relationship. Findings suggest that being isolated in later life is detrimental to cognitive health. We also find that cognitive reserve moderates this association at two year follow-up.

The finding that social isolation is associated with cognitive function at baseline and two year follow-up is consistent with previous studies that assess these relationships using baseline [25] and longitudinal data [26–28]. This suggests that being socially integrated in later life is beneficial to cognitive function. Social isolation may be detrimental to cognitive function as isolated individuals experience less social contact with others. Such individuals are likely to receive less cognitive stimulation through social contact, resulting in lower cognitive reserve and hence poorer cognitive function [8, 9].

We also found that the relationship between social isolation and cognition is moderated by cognitive reserve longitudinally, but not cross-sectionally. This suggests that having higher cognitive reserve further benefits late-life cognitive function. Cognitive reserve may explain the differences in cognitive trajectories observed in later life [8]. When each aspect of the cognitive reserve measure was separated in moderation analyses, we found that education and cognitive activity did not moderate the association, but occupational complexity did. Further analysis shows that the association of social isolation with cognitive change was significant in individuals with low occupational complexity, but not high occupational complexity. This suggests that good social interactions may be more beneficial to cognition in individuals with low mid-life reserve, as measured by occupational complexity [27, 41]. Similar findings have been reported for other health outcomes. For instance, participation in social activities has been associated with better health among participants from low socio-economic groups [42]. Likewise, it has been found that good social resources benefit health outcomes for those in low-income groups [43]. This provides further support for the beneficial effect of social connections on individuals with low mid-life reserve [27, 43].

Education alone did not moderate the association between isolation and cognitive function. This is inconsistent with a previous study by Shankar and colleagues [27] which reported that education did moderate this association. However, the authors of this study assessed three sub-domains of cognition, and found that education moderated the association between isolation and cognition for delayed recall, but not for verbal fluency or immediate recall [27]. In addition, the education measure in the present study is a relatively crude index and only considers the number of years of education. Shankar and colleagues [27] measure education based on the highest qualification obtained, and classify participant’s educational level as either low (no formal qualifications) or high. This measure of education is much more sensitive. This reflects how methodological differences across studies may be accountable for differences in findings.

Cognitive ability can be improved across the lifespan based on lifestyle and engagement [44]. This has important implications for preventative interventions. Although some aspects of early- and mid- life reserve cannot be modified in later life, such as education or occupational complexity, there are other aspects of reserve that can be modified, such as social activity, to contribute to building reserve and reducing poor cognitive function in later life. This may be particularly useful for individuals that have poor early- and mid- life reserve. The results of the present study reflect that social isolation in later life is detrimental to cognitive function. Interventions to reduce social isolation in older people may benefit cognitive function both directly and indirectly through building cognitive reserve.

This study has many strengths, including the use of a large scale, population-based cohort study. Participants in CFAS-Wales were sampled from general practice registers and approached to participate, giving a representative sample of older people, including those who are extremely isolated. Studies that acquire samples through participants responding to advertised studies may be more at risk of bias to a ‘self-selected’ sample that are more engaged in social and community activities, and hence are not isolated. A second advantage is that the cognitive reserve measure combined several proxy indicators from early- (education), mid- (occupational complexity), and late- (cognitive activity) life. This approach is preferable as it accounts for the variance in reserve built at different stages of life [30, 31]. Each of these components was weighted equally to generate a cognitive reserve score. However, the contribution of each of these components of the cognitive reserve score may vary considerably across participants. For example, an individual with a poor education and low occupational complexity may compensate for this through good social relationships [27]. These individual differences may not be adequately reflected in the measure.

This study also has some limitations. It is possible that social isolation may be prodromal to poor cognitive function [45]. However, we excluded participants with poor cognitive function or dementia at baseline which reduces the risk of reverse causation. In addition, the same association was found between social isolation and cognitive function at baseline and two year follow-up. An additional limitation is that very little cognitive change was observed across the sample between baseline and follow-up. Some participants had improved on their CAMCOG scores at follow-up. This limits the validity of the longitudinal analyses as they may replicate the cross-sectional findings, rather than reliably representing the longitudinal relationship between isolation and cognitive change. The follow-up period of two years may not be sufficient to observe cognitive change.

The findings of this study suggest that being socially isolated in later life is associated with poor cognitive function. This has important implications for interventions, suggesting that targeting isolation may be beneficial for cognitive health. The finding that cognitive reserve moderates this association reflects the importance of being engaged throughout the lifespan in order to build reserve to protect against poor cognitive function in later life.

## Supporting information

S1 Table Comparison of included and excluded participants at two year follow-up. S1 Table provides a comparison of participants who were included and excluded at follow-up. Excluded participants were significantly older, had a significantly lower baseline CAMCOG score, fewer years of education, lower scores for cognitive activity, lower occupational complexity, lower cognitive reserve score, lower LSNS-6 score, poorer eyesight, and required significantly more help with daily tasks.
(DOCX)

S2 Table Cross-sectional association between social isolation and sub-domains of cognition assessed by the CAMCOG. We conducted further regression analyses to determine whether social isolation was more associated with any specific cognitive domains assessed by the CAMCOG (see “S2 Table”). Social isolation was significantly associated with orientation (adjusted R^2^ = .02, *F*(7, 2216) = 6.23, *p* < .001), expression (adjusted R^2^ = .13, *F*(7, 2216) = 48.30, *p* < .001), praxis (adjusted R^2^ = .06, *F*(7, 2216) = 21.60, *p* < .001), and perception (adjusted R^2^ = .11, *F*(7, 2216) = 42.04, *p* < .001), but not with comprehension (adjusted R^2^ = .02, *F*(7, 2216) = 7.11, *p* < .001), memory (adjusted R^2^ = .04, *F*(7, 2216) = 14.60, *p* < .001), attention and calculation (adjusted R^2^ = .03, *F*(7, 2216) = 9.36, *p* < .001), or abstract thinking (adjusted R^2^ = .05, *F*(7, 2216) = 16.80, *p* < .001).
(DOCX)

S3 Table Longitudinal association between social isolation and cognitive change. A sensitivity analysis was conducted to determine the reliability of the regression analysis assessing the relationship between social isolation and cognitive change score.
As there was little cognitive change across the sample over two years, a binary variable was created to distinguish cognitive decliners from non-decliners. Decliners were defined as a decline in follow-up CAMCOG score of one standard deviation unit from the baseline CAMCOG score. In total, 203 (13%) participants were classified as cognitive decliners and 1,321 (87%) of participants maintained good cognitive function.
A logistic regression was conducted to assess whether there was an association between social isolation and cognitive decline over the two year follow-up (“S3 Table”). The logistic regression model was significant suggesting that social isolation was associated with a decline in CAMCOG score over two years, X^2^(1) = 8.20, *p* = .004. This remained significant after controlling for covariates, X^2^(7) = 88.80, *p* < .001. This suggests that people who are less socially isolated have a small reduction in risk of cognitive decline over a two year follow-up, whereas people who are isolated have a greater risk of cognitive decline.
(DOCX)

S4 Table Longitudinal association between social isolation and sub-domains of cognition assessed by the CAMCOG. We conducted further regression analyses to determine whether social isolation was more associated with any specific cognitive domains assessed by the CAMCOG using longitudinal data (see “S4 Table”). Social isolation was significantly associated with comprehension (adjusted R^2^ = .01, *F*(7, 1516) = 2.14, *p* = .037), but not with orientation (adjusted R^2^ = .01, *F*(7, 1516) = 2.74, *p* = .008), expression (adjusted R^2^ = 0, *F*(7, 1516) = 1.72, *p* = .099), memory (adjusted R^2^ = .04, *F*(7, 1516) = 9.06, *p* < .001), attention and calculation (adjusted R^2^ = .01, *F*(7, 1516) = 2.95, *p* = .005), praxis (adjusted R^2^ = 0, *F*(7, 1516) = 1.95, *p* = .059), abstract thinking (adjusted R^2^ = .01, *F*(7, 1516) = 2.77, *p* = .007), and perception (adjusted R^2^ = .01, *F*(7, 1516) = 1.44, *p* = .184).
(DOCX)
