# Supplementary material for: Social isolation, cognitive reserve, and cognition in healthy older people
Source: PLoS One. 2018 Aug 17;13(8):e0201008. doi: 10.1371/journal.pone.0201008 (PMC6097646; doi:10.1371/journal.pone.0201008)
Supplement: S1 Table — provides a comparison of participants who were included and excluded at follow-up. Excluded participants were significantly older, had a significantly lower baseline CAMCOG score, fewer years of education, lower scores for cognitive activity, lower occupational complexity, lower cognitive reserve score, lower LSNS-6 score, poorer eyesight, and required significantly more help with daily tasks. (DOCX) [file pone.0201008.s001.docx]

**S1: Comparison of included and excluded participants at two year follow-up**

“S1 Table” provides a comparison of participants who were included and excluded at follow-up. Excluded participants were significantly older, had a significantly lower baseline CAMCOG score, fewer years of education, lower scores for cognitive activity, lower occupational complexity, lower cognitive reserve score, lower LSNS-6 score, poorer eyesight, and required significantly more help with daily tasks.

S1 Table. Comparison of included and excluded participants at two year follow-up

| **Variable** | **Included participants**  **(N = 1,524)** | **Excluded participants**  **(N = 700)** | ***t*(df) or X^2^(df),**  ***p*** |
| --- | --- | --- | --- |
| Age (years), *M* (SD) | 73.23 (6.14) | 73.99 (6.54) | *t*(1, 2222) = 2.67,  *p* = .007 |
| Gender, *N* (%) |  |  |  |
| Men | 758 (49.74) | 339 (48.43) | X^2^(1) = .33, |
| Women | 766 (50.26) | 361 (51.57) | *p* = .57 |
| Baseline CAMCOG score, *M* (SD) | 94.17 (4.95) | 92.01 (5.88) | *t*(1, 2222) = -8.93,  *p* < .001 |
| Education (years), *M* (SD) | 12.18 (2.83) | 11.77 (2.66) | *t*(1, 2222) = -3.25,  *p* < .001 |
| Cognitive activity, *M* (SD) | 21.57 (5.15) | 20.81 (5.23) | *t*(1, 2222) = -3.22,  *p* < .001 |
| Occupation complexity, *M* (SD) | 8.34 (3.30) | 7.59 (3.32) | *t*(1, 2222) = -4.98,  *p* < .001 |
| Cognitive reserve score, *M* (SD) | 61.34 (11.46) | 58.86 (11.04) | *t*(1, 2222) = -5.36,  *p* < .001 |
| LSNS-6, *M* (SD) | 16.43 (5.79) | 15.49 (5.64) | *t*(1, 2222) = -3.58,  *p* < .001 |
| Health conditions, *N* (%)^a^ |  |  |  |
| Hearing | 429 (28.15) | 200 (28.57) | X^2^(1) = .04,  *p* = .84 |
| Eyesight | 192 (12.60) | 119 (17.00) | X^2^(1) = 7.73,  *p* = .005 |
| Require help with daily tasks, *N* (%) | 400 (26.25) | 237 (33.86) | X^2^(1) = 13.59,  *p* < .001 |

Notes: ^a^ Number and percentage of people who have these health conditions and rate these conditions as physically limiting.
