# Supplementary material for: Social isolation, cognitive reserve, and cognition in healthy older people
Source: PLoS One. 2018 Aug 17;13(8):e0201008. doi: 10.1371/journal.pone.0201008 (PMC6097646; doi:10.1371/journal.pone.0201008)
Supplement: S2 Table — We conducted further regression analyses to determine whether social isolation was more associated with any specific cognitive domains assessed by the CAMCOG (see “S2 Table”). Social isolation was significantly associated with orientation (adjusted R2 = .02, F(7, 2216) = 6.23, p < .001), expression (adjusted R2 = .13, F(7, 2216) = 48.30, p < .001), praxis (adjusted R2 = .06, F(7, 2216) = 21.60, p < .001), and perception (adjusted R2 = .11, F(7, 2216) = 42.04, p < .001), but not with comprehension (adjusted R2 = .02, F(7, 2216) = 7.11, p < .001), memory (adjusted R2 = .04, F(7, 2216) = 14.60, p < .001), attention and calculation (adjusted R2 = .03, F(7, 2216) = 9.36, p < .001), or abstract thinking (adjusted R2 = .05, F(7, 2216) = 16.80, p < .001). (DOCX) [file pone.0201008.s002.docx]

**S2: Cross-sectional association between social isolation and sub-domains of cognition assessed by the CAMCOG**

“S2 Table”. Cross-sectional association between social isolation and sub-domains of cognition assessed by the CAMCOG

|  | | | | | | | | |
| --- | --- | --- | --- | --- | --- | --- | --- | --- |
|  | **Orientation** | **Comprehension** | **Expression** | **Memory** | **Attention and calculation** | **Praxis** | **Abstract thinking** | **Perception** |
|  | *B* (95% CI)  *p* | *B* (95% CI)  *P* | *B* (95% CI)  *p* | *B* (95% CI)  *p* | *B* (95% CI)  *p* | *B* (95% CI)  *p* | *B* (95% CI)  *p* | *B* (95% CI)  *p* |
| **Social isolation** | .01 (0, .02)  .003 | 0 (-.02, .01)  .514 | .02 (.01, .03)  .002 | .02 (0, .03)  .072 | .01 (0, .03)  .109 | .03 (.01, .05)  <.001 | 0 (-.02, .03)  .809 | .05 (.02, .09)  .002 |
| **Age** | 0 (0, 0)  .413 | 0 (-.01, 0)  <.001 | -.01, (-.02, -.01)  <.001 | -.01 (-.01, 0)  <.001 | 0 (-.01, 0)  .043 | -.01 (-.01, -.01)  <.001 | -.01 (-.01, -.01)  <.001 | -.04 (-.04, -.03)  <.001 |
| **Gender** | 0 (-.01, .02)  .807 | .03 (0, .05)  .059 | .02 (-.01, .04)  .199 | -.07 (-.10, -.03)  <.001 | -.09 (-.12, -.06)  <.001 | -.07 (-.11, -.03)  <.001 | .05 (0, .09)  .045 | -.07 (-.14, -.01)  .025 |
| **Education** | 0 (0, .01)  <.001 | .01 (0, .01)  .003 | .02 (.02, .02)  <.001 | .01 (.01, .02)  <.001 | .01 (.01, .02)  <.001 | .02 (.01, .02)  <.001 | .03 (.02, .04)  <.001 | .02 (.01, .03)  <.001 |
| **Eyesight** | .02 (0, .04)  .041 | .01 (-.03, .05)  .643 | -.01 (-.04, .03)  .742 | -.05 (-.10, -.01)  .026 | -.02 (-.07, .02)  .301 | -.07 (-.12, -.02)  .009 | -.13 (-.20, -.07)  <.001 | -.09 (-.19, 0)  .055 |
| **Hearing** | 0 (-.02, .02)  .996 | -.01 (-.04, .02)  .376 | -.01 (-.04, .02)  .491 | -.04 (-.08, -.01)  .023 | .01 (-.03, .05)  .588 | -.06 (-.10, -.02)  .003 | -.01 (-.05, .04)  .826 | -.05 (-.13, .02)  .146 |
| **Help with daily activity** | -.03 (-.04, -.01)  <.001 | -.04 (-.07, -.01)  .013 | -.03 (-.06, 0)  .021 | -.02 (-.05, .02)  .372 | -.01 (-.05, .02)  .476 | -.04 (-.08, .0)  .065 | -.01 (-.06, .04)  .777 | -.06 (-.14, .01)  .092 |

We conducted further regression analyses to determine whether social isolation was more associated with any specific cognitive domains assessed by the CAMCOG (see “S2 Table”). Social isolation was significantly associated with orientation (adjusted R^2^ = .02, *F*(7, 2216) = 6.23, *p* <.001), expression (adjusted R^2^ = .13, *F*(7, 2216) = 48.30, *p* <.001), praxis (adjusted R^2^ = .06, *F*(7, 2216) = 21.60, *p* <.001), and perception (adjusted R^2^ = .11, *F*(7, 2216) = 42.04, *p* <.001), but not with comprehension (adjusted R^2^ = .02, *F*(7, 2216) = 7.11, *p* <.001), memory (adjusted R^2^ = .04, *F*(7, 2216) = 14.60, *p* <.001), attention and calculation (adjusted R^2^ = .03, *F*(7, 2216) = 9.36, *p* <.001), or abstract thinking (adjusted R^2^ = .05, *F*(7, 2216) = 16.80, *p* <.001).
