# Supplementary material for: Social isolation, cognitive reserve, and cognition in healthy older people
Source: PLoS One. 2018 Aug 17;13(8):e0201008. doi: 10.1371/journal.pone.0201008 (PMC6097646; doi:10.1371/journal.pone.0201008)
Supplement: S3 Table — A sensitivity analysis was conducted to determine the reliability of the regression analysis assessing the relationship between social isolation and cognitive change score. As there was little cognitive change across the sample over two years, a binary variable was created to distinguish cognitive decliners from non-decliners. Decliners were defined as a decline in follow-up CAMCOG score of one standard deviation unit from the baseline CAMCOG score. In total, 203 (13%) participants were classified as cognitive decliners and 1,321 (87%) of participants maintained good cognitive function. A logistic regression was conducted to assess whether there was an association between social isolation and cognitive decline over the two year follow-up (“S3 Table”). The logistic regression model was significant suggesting that social isolation was associated with a decline in CAMCOG score over two years, X2(1) = 8.20, p = .004. This remained significant after controlling for covariates, X2(7) = 88.80, p < .001. This suggests that people who are less socially isolated have a small reduction in risk of cognitive decline over a two year follow-up, whereas people who are isolated have a greater risk of cognitive decline. (DOCX) [file pone.0201008.s003.docx]

**S3: Longitudinal association between social isolation and cognitive change**

A sensitivity analysis was conducted to determine the reliability of the regression analysis assessing the relationship between social isolation and cognitive change score.

As there was little cognitive change across the sample over two years, a binary variable was created to distinguish cognitive decliners from non-decliners. Decliners were defined as a decline in follow-up CAMCOG score of one standard deviation unit from the baseline CAMCOG score. In total, 203 (13%) participants were classified as cognitive decliners and 1,321 (87%) of participants maintained good cognitive function.

A logistic regression was conducted to assess whether there was an association between social isolation and cognitive decline over the two year follow-up (“S3 Table”). The logistic regression model was significant suggesting that social isolation was associated with a decline in CAMCOG score over two years, X^2^(1) = 8.20, *p* = .004. This remained significant after controlling for covariates, X^2^(7) = 88.80, *p* < .001. This suggests that people who are less socially isolated have a small reduction in risk of cognitive decline over a two year follow-up, whereas people who are isolated have a greater risk of cognitive decline.

“S3 Table”. Longitudinal association between social isolation and cognitive change

|  | **Model 1**  OR (95% CI)  *p* | **Model 2**  OR (95% CI)  *p* | **Model 3**  OR (95% CI)  *p* |
| --- | --- | --- | --- |
| **Social isolation** | .80 (.69, .93)  .004 | .85 (.73, 1.00)  .05 | .85 (.73, 1.00)  .05 |
| **Age** | - | 1.10 (1.07, 1.12)  < .001 | 1.10 (1.08, 1.13)  < .001 |
| **Gender** | - | 1.30 (.95, 1.77)  .10 | 1.33 (.97, 1.82)  .08 |
| **Education** | - | .93 (.88, .99)  .02 | .93 (.87, .99)  .02 |
| **Eyesight** | - | - | 1.06 (.69, 1.65)  .78 |
| **Hearing** | - | - | .76 (.53, 1.08)  .13 |
| **Help with daily activity** | - | - | .74 (.51, 1.06)  .10 |

Notes: OR = odds ratio, CI = confidence interval. Model 1: unadjusted; Model 2: adjusted for age, gender, and years of education; Model 3: adjusted for age, gender, education, physically limiting health conditions (eyesight and hearing), and help with daily activities.
